# Bibliometric analysis of quality of life in implant-based breast reconstruction

**DOI:** 10.3389/fonc.2024.1429885

**Published:** 2024-08-08

**Authors:** Kian Daneshi, Francesca Ruccia, Radhika Merh, Tommaso Barlattani, Raed Alderhalli, Mark Warren Clemens, Ankur Khajuria

**Affiliations:** ^1^ School of Medicine and Population Health, The University of Sheffield, Sheffield, United Kingdom; ^2^ Department of Plastic and Reconstructive Surgery, The Royal Marsden NHS Foundation Trust, London, United Kingdom; ^3^ Department of Breast Surgery, The Royal Marsden NHS Foundation Trust, London, United Kingdom; ^4^ Department of Biotechnological and Applied Clinical Sciences, University of L’Aquila, L’Aquila, Italy; ^5^ Department of Medicine, Newcastle University Medicine Malaysia, Johor, Malaysia; ^6^ Department of Plastic Surgery, The University of Texas MD Anderson Cancer Center, Houston, TX, United States; ^7^ Kellogg College, University of Oxford, Oxford, United Kingdom; ^8^ Department of Surgery & Cancer, Imperial College London, London, United Kingdom

**Keywords:** breast reconstruction, quality of life, patient-reported outcome, breast implants, Web of Science, psychological wellbeing, patient satisfaction, BREAST-Q

## Abstract

**Background:**

Implant-based breast reconstruction (IBR), following mastectomy, significantly impacts patients’ quality of life (QoL), necessitating accurate measurement through psychometrically robust patient-reported outcome measure (PROM) tools. This bibliometric analysis aims to discern trends, identify gaps, and evaluate the use of such tools in the IBR literature.

**Methods:**

The 100 most cited publications regarding QoL in implant-based reconstruction were identified on Web of Science, across all available journal years (from 1977 to 2024) on 10 March 2024. Study details, including the citation count, main content focus, outcome measures, and usage of psychological questionnaires, were extracted and tabulated from each publication. The Oxford Centre for Evidence-Based Medicine (OCEBM) levels of evidence (LOE) of each study were assessed.

**Results:**

The 100 most cited publications on QoL in implant-based reconstruction were identified, encompassing 64,192 patients and 28,114 reconstructed breasts. Citations per publication ranged from 62 to 457 (mean, 124.95 ± 73.05), with the highest-cited study being authored by Al-Ghazal (n = 457). The vast majority of publications were LOE II (n = 52), representative of prospective cohort studies, systematic reviews of non-randomised studies, and systematic review and meta-analysis. The number of publications for LOE V, IV, III, and I was 0, 7, 41, and 0, respectively. The main content focus was “quality of life” in 83 publications, with significant utilisation of the BREAST-Q questionnaire. A total of 80 publications used validated questionnaires with psychometric development.

**Conclusions:**

This analysis demonstrates that the research methodologies within IBR mostly consist of moderate-quality publications; however, notably, there was a lack of LOE I studies, underscoring a gap in high-quality research within the field. Moreover, only 62/100 used validated PROM tools. Future IBR research studies should be focussed on most robust methodologies, incorporating validated PROM tools, to optimise shared-decision making and informed consent.

## Introduction

Implant-based breast reconstruction (IBR) restores the breast contour following mastectomy, with various techniques ranging from Direct-to-Implant Reconstruction (DTI) to Two-Stage Reconstruction with Tissue Expanders (1, 2). IBR not only improves the physical proportions of patients post-mastectomy, but also additionally restores and sometimes elevates, from baseline, the patient’s psychological confidence, and thus their quality of life (QoL) (3–5). Advancements in techniques have shaped patient expectations and aesthetic standards for breast appearance. Whilst traditional clinical outcome measurement is necessary, it is no longer sufficient (6). Measuring patient-reported outcomes (PROs) assessing physical, psychosexual, and social wellbeing and overall QoL is paramount for optimal outcome assessment, with the utilisation of validated tools such as the BREAST-Q, given the significant impact the implications of requiring IBR and undergoing the procedure can have on one’s QoL and bodily self-esteem (7, 8).

Bibliometric analyses have the capacity to scrutinise vast data sets and leverage citation metrics to pinpoint research trends and identify gaps in the literature. In the context of IBR, two previous bibliometric analyses have been completed, following global research on breast reconstruction post-mastectomy and research trends within breast reconstruction (9, 10). Both studies covered both IBR and autologous breast reconstruction. A major drawback of both studies is their lack of discussion and assessment of patient-reported outcome measures (PROMs) and, by extension, QoL, which are a paramount tenet of the reconstructive breast surgery outcome set (11). Given this gap within the literature, our unique bibliometric analysis aims to provide an objective evaluation and discussion regarding QoL within patients who have experienced IBR.

We conducted the first comprehensive bibliometric analysis of articles published on IBR, to evaluate the quality and characteristics of the top 100 cited articles, and to highlight key findings from cited articles on QoL measures. We hypothesise that amongst the top 100 cited articles on IBR, there is a predominance of lower-level evidence research and a notable scarcity of studies assessing psychological wellbeing and PROs—a trend observed in previous bibliometric analyses within reconstructive breast surgery.

## Methods

A comprehensive literature review was conducted to identify the 100 most cited publications on IBR. We searched all journal publications available on the Web of Science online database (Clarivate Analytics, Philadelphia, PA), with the search strategy being available as a **Supplementary Table 1**. The search terms inputted appeared as a “topic” and was completed on 10 March 2024. The time span included all available years (1977–2024). The criteria for inclusion were publications in journals from this research strategy. Exclusion criteria were publications in non-English language, papers not focussed on breast reconstruction with implants, not pertaining to QoL measures as at least one of their studied outcomes, animal studies, other surgical procedures, and duplicate publications. The LOE was assessed in accordance with the Oxford Centre for Evidence-Based Medicine (OCEBM) system (12).

**Figure 1:**
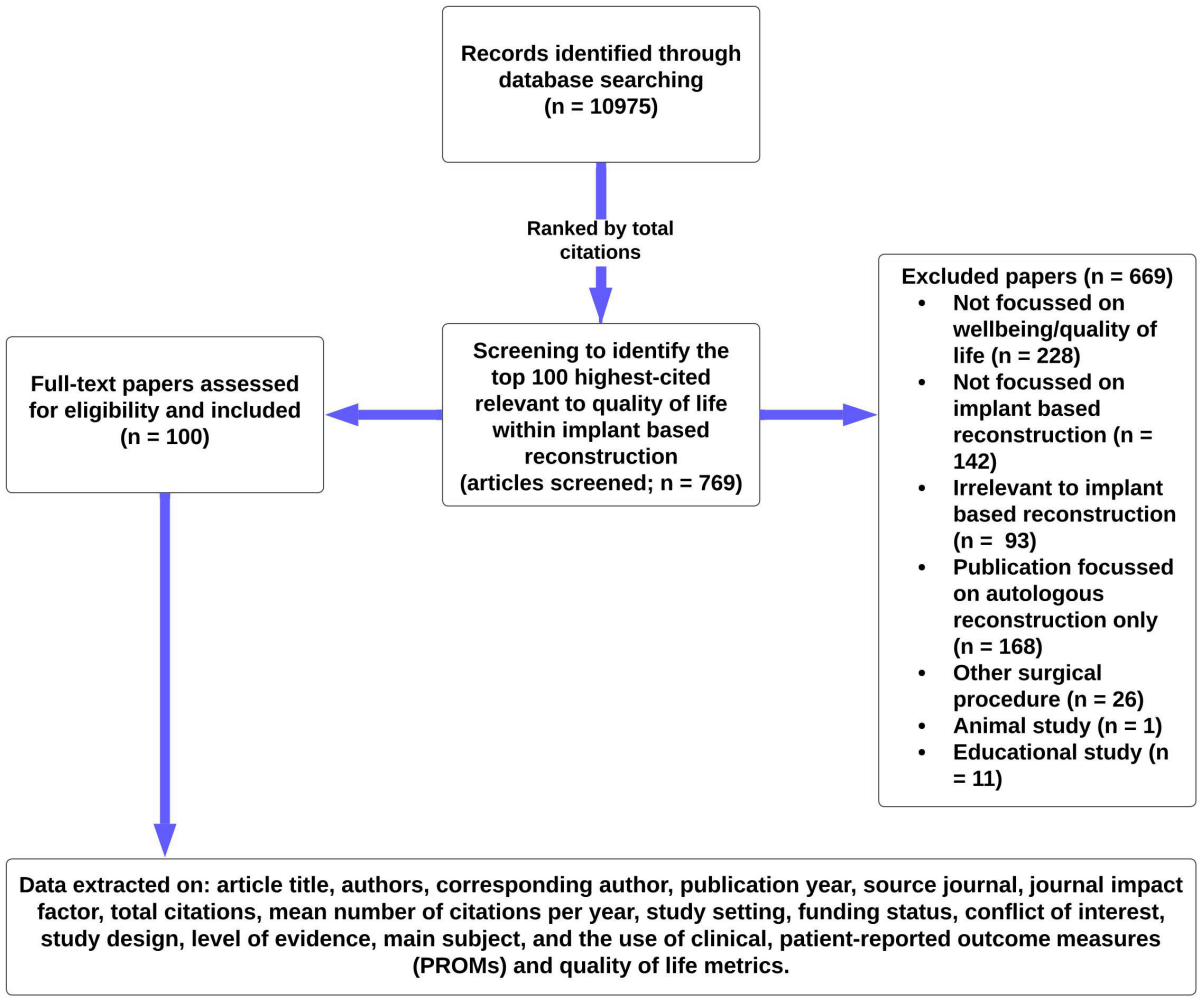
Summary flowchart of methodology.

The search yielded a total of 10,975 publications in journals, which were then ranked according to the number of citations. Publications with the same amount of citations were separated according to the mean number of citations per year; if the average number of citations per year was equal, they were sorted based on the journal’s impact factor. To guarantee that all publications were directly relevant to IBR, two reviewers (KD and FR) independently screened titles and abstracts independently until 100 journal publications were included. Any discrepancies that could not be resolved between KD and FR were solved by consensus-based discussion with the third (RM) and senior author (AK), in addition to ratification by reviewing the full text of the publication. A total of 769 journal papers were screened to obtain the 100 most frequent publications for inclusion. Reasons for other publication exclusions are specified in **Figure 1**.

Data were extracted from full-text publications in a standardised online spreadsheet (Google Sheets: Google LLC, Mountain View, California, USA). Data retrieval included publication title, list of authors, corresponding author, year of publication, journal of origin, total citations, the average number of citations per year, geographic context of the study, source of funding, study design, LOE, main topic/content focus, conflict of interest (CoI) statement, and use of validated clinical, psychological, cosmetic, and PROMs.

## Results

### Citation analysis

The 100 most cited publications regarding patient satisfaction and psychological wellbeing of patients who have had implant-based reconstruction were cited by 12,495 publications. The number of citations accrued per paper ranged from 62 to 457. Publications were cited with a mean of 124.95 times ± 73.05. The mean number of citations per publication per year ranged from 2.69 to 34.86 (mean, 9.13) (**Supplementary Table 2**).

#### Authorship

The highest cited publication (*n* = 457), authored by Al-Ghazal, SK et al., was published in 2000 and reviewed, assessed, and compared the psychological outcome and satisfaction of patients who underwent wide local excision, mastectomy alone, and mastectomy with breast reconstruction (13). The most prolific corresponding author was Pusic, AL with six publications as corresponding author, followed by Cordeiro, PG, Lee, BT, and Wilkins, EG at four each, and Alderman, AK at three publications. Active publications were counted next, with values for authors being calculated by the frequency of first authorships. Cordeiro was the author with the highest number of first authorships with four; Pusic had the second highest (*n* = 3); however, she had five additional publications as second author (**Supplementary Table 2**).

#### Centre, time frame, and funding

**Figure 2:**
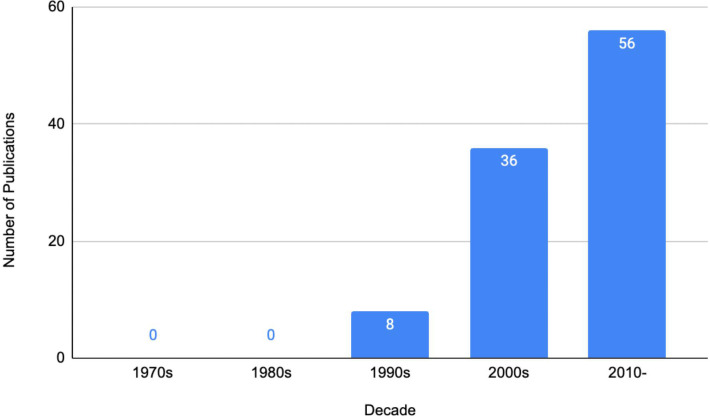
The 100 most cited publications—a by-decade analysis.

Single-centre studies represented the majority of the top 100 publications (*n* = 81); there were five multicentre studies, mostly between the USA and Canada. In total, 17 individual countries contributed to the 100 most cited publications, with the USA contributing to almost half of the 100 (*n* = 45) publications; however, the total number of publications associated with the USA is higher given their involvement with numerous multi-centre studies in addition to single-centre studies (**Table 1**). A significant portion of the most frequently cited publications were published in the 2000s and specifically 2010 onwards, coming at 36 and 56 publications, respectively, with the three most cited publications being published in the year 2000 (**Figure 2**).

**Table 1:** Country frequency in the 100 most cited IBR publications.

Rank	Country	Number of publications
1	USA	45
2	Multicentre	19
3	UK	10
4	Netherlands	5
5	Sweden	3
6	Australia	3
7	Canada	2
8	France	2
9	Italy	2
10	Spain	2
11	Belgium	1
12	Denmark	1
13	Greece	1
14	Ireland	1
15	Israel	1
16	Norway	1
17	Taiwan	1

A total of 38 studies formally acknowledged the receipt of funding; a smaller proportion of funding (*n* = 5) was provided by various private entities such as Allergan, Abbvie, and the Lifecell Corporation. Five publications stated the receipt of a grant from a university or university-based faculty leadership. The remaining funding sources included the National Institutes of Health (NIH), the National Cancer Institute (NCI), the Plastic Surgery Foundation, and joint ventures between governmental or state departments with universities and various charitable and private corporations. A total of 38 studies explicitly stated receipt of no funding, and the remaining 24 studies did not specify whether they received funding or not from external (governmental, industry, institutional, etc.) or internal (departmental and divisional levels of organisations) sources. Additionally, four publications disclosed potential CoI with most authors stating that they were consultants or held equity in and for the companies providing products used. A further 68 publications explicitly stated that there were no CoIs to declare, and the remaining 26 did not specify if there was any CoI or not.

#### Journals

The 100 most cited publications on patient satisfaction and psychological wellbeing in IBR originated from 30 journals. *Plastic and Reconstructive Surgery* was the most popular journal (*n* = 39). A majority (*n* = 51) of publications were published in non-plastic surgery dedicated journals such as *Annals of Surgery, The Breast*, and *Quality of Life Research* (**Supplementary Table 3**). The journal with the highest impact factor present was *Annals of Oncology* (*n* = 50.5), which contributed one publication to the top 100 (see **Supplementary Table 3** for the list of the journals present).

#### Research themes

QoL/PRO was the main subject or primary study outcome in the vast majority of publications (*n* = 83), with many publications utilising the validated BREAST-Q scale (**Supplementary Table 3**) (7). A total of 20 studies categorically did not use any validated PRO questionnaires to assess QoL, and 14 studies used a combination of validated and non-validated questionnaires.

Various types of mediums were used to assess PROs, with many studies utilising multiple questionnaires; the top five most used PROs/questionnaires were as follows: “Self-assessment/customised questionnaire”, “BREAST-Q”, “Body Image after Breast Cancer Questionnaire” (BIBCQ), “Functional Assessment of Cancer Therapy - Breast” (FACT-B), and “EORTC QLQ-BR23” with 69, 38, 12, 10, and 9 occurrences, respectively. Other prevalent research themes included clinical outcomes and surgical technique, seen in 12 and 5 publications, respectively. The BREAST-Q was the most frequently used validated questionnaire (*n* = 38). In total, validated questionnaires were used 178 times and non-validated questionnaires were utilised on 69 occasions. See **Supplementary Table 4** for a breakdown of all the questionnaires utilised and the frequency of their usage.

**Figure 3:**
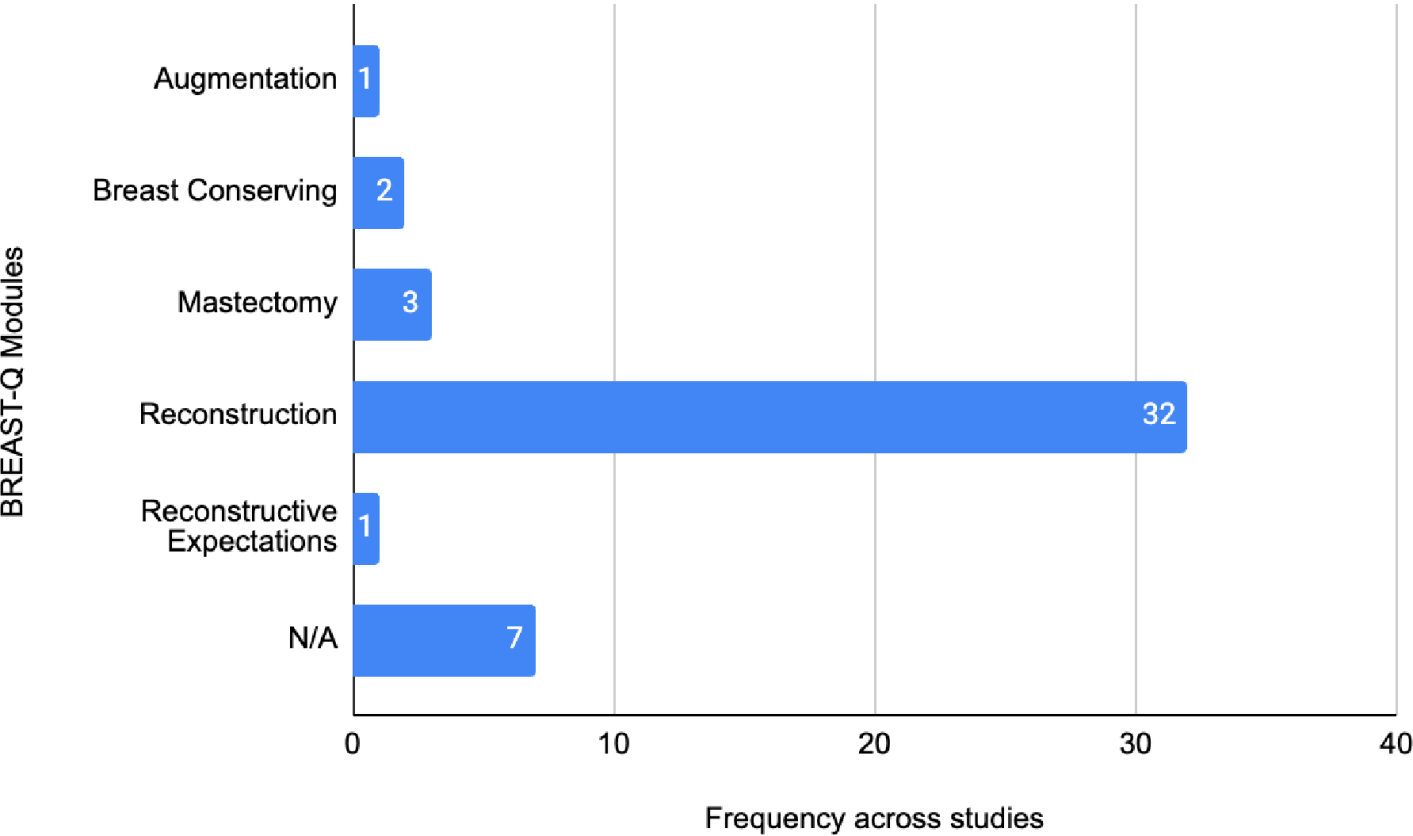
Frequency of BREAST-Q modules across IBR studies.

In our study, we further assessed the studies utilising the BREAST-Q, by analysing the proportions between the usage of the subsequent BREAST-Q modules: Augmentation, Reduction/Mastopexy, Mastectomy, Reconstruction, Reconstruction Expectations, and Breast Conserving Therapy. A total of 38 studies utilised the BREAST-Q; however, numerous studies incorporated multiple modules. “Augmentation” was used on one occasion, “Breast Conserving Therapy” appeared twice, “Mastectomy” appeared thrice, “Reconstruction” appeared 31 times, “Reconstruction Expectations” appeared 1 time, and 7 studies did not specify which module they used; however, one could infer that they most likely all used “Reconstructive”. See **Figure 3** for a breakdown.

Most publications’ main content focus was “Quality of life/PRO” (*n* = 83), followed by “Clinical outcomes” (*n* = 12) and “Surgical technique” (*n* = 5). It is important to distinguish studies that still discussed or mentioned clinical outcomes, but although they were not the main focus of the content, they were still tabulated (**Figure 3**). A total of 34 publications discussed clinical outcomes; this is inclusive of the 12 where it was the main subject focus. Of these studies, 27 examined clinical outcomes and discussed “implant-related complications”, and 8 discussed “major complications” and discussed “flap-related complications”.

#### Methodological quality

**Figure 4:**
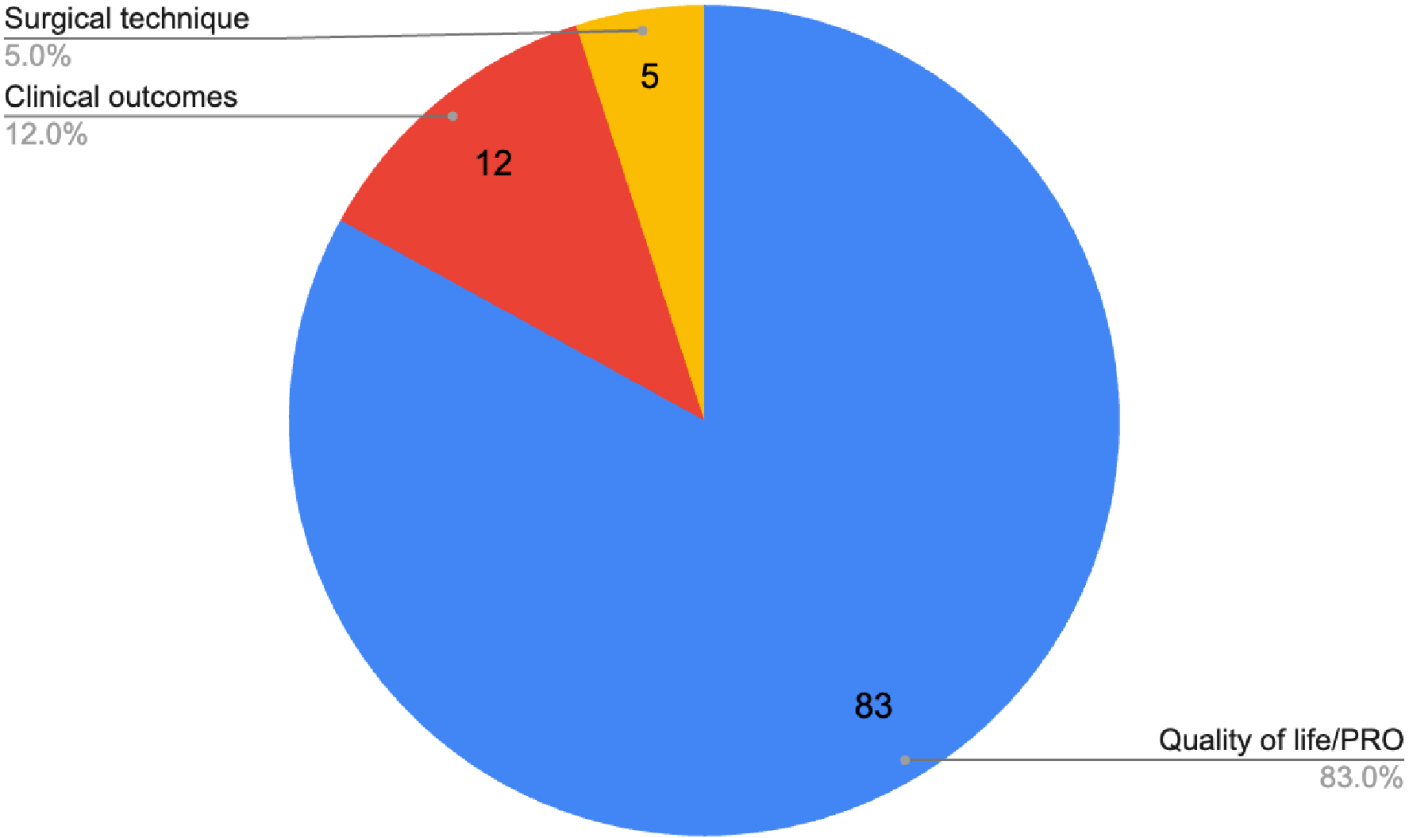
Main subjects of the 100 most cited publications on IBR.

**Figure 5:**
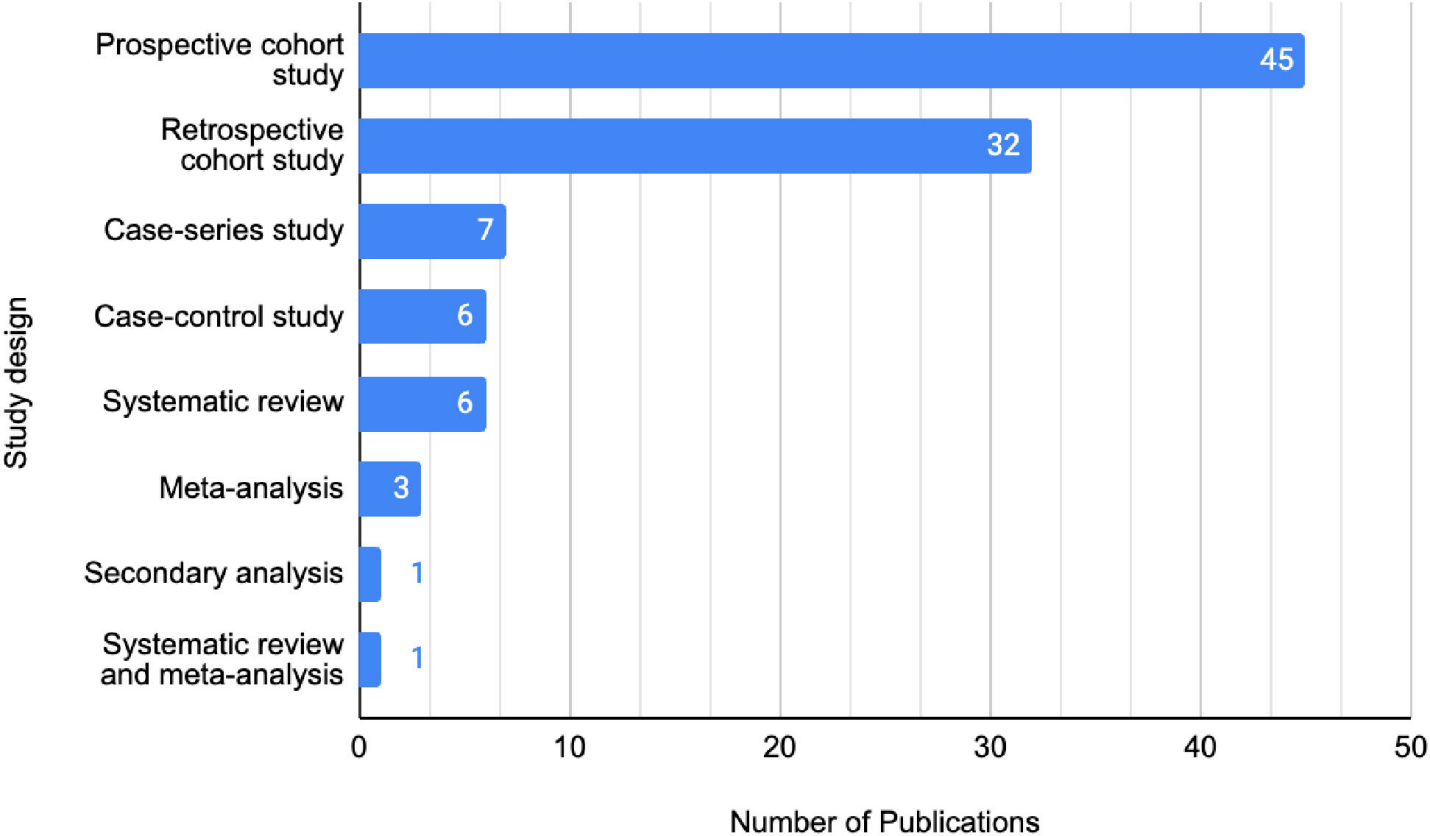
Study designs of the 100 most cited publications on IBR.

**Figure 6:**
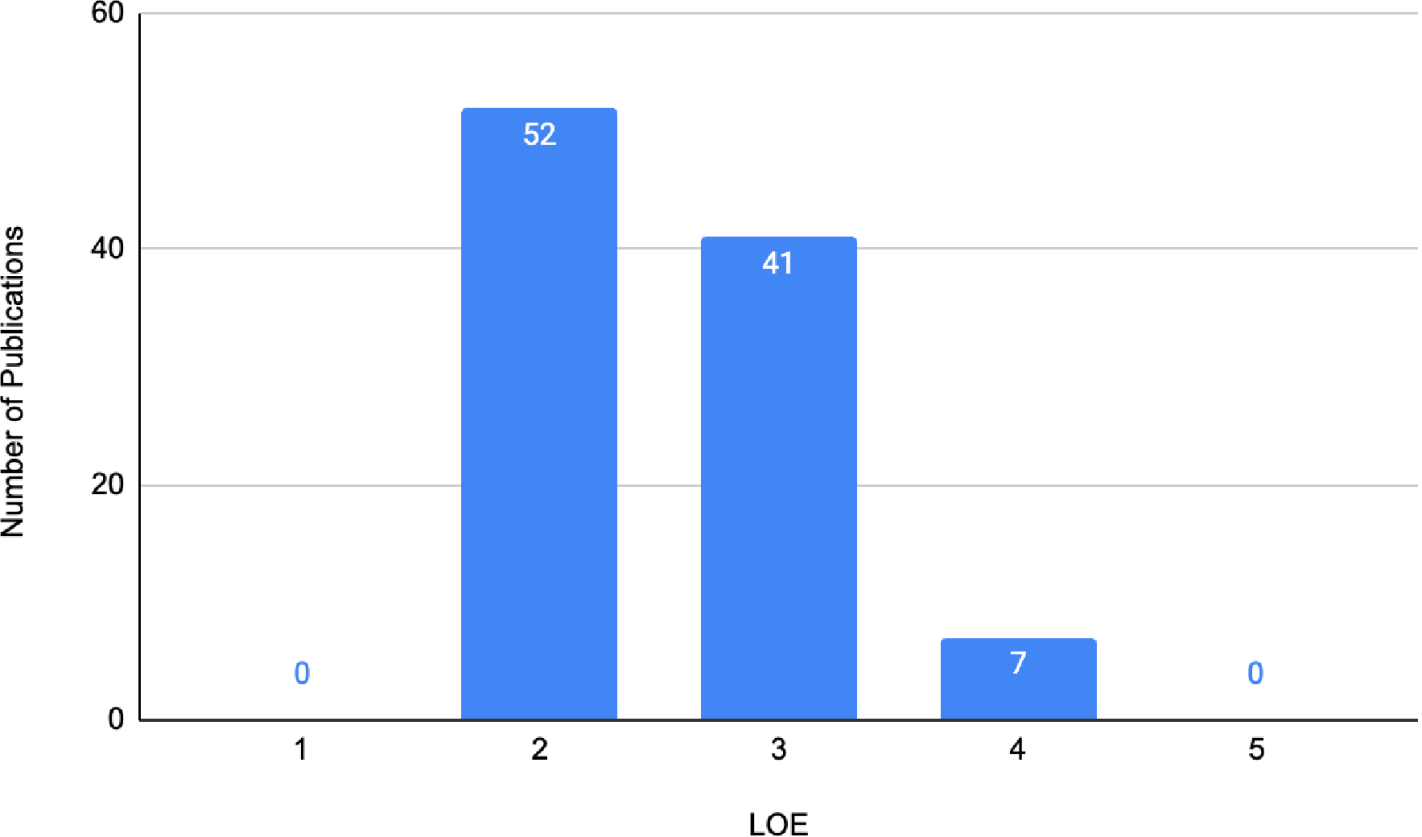
Levels of evidence for the 100 most cited studies on IBR.

**Figure 7:**
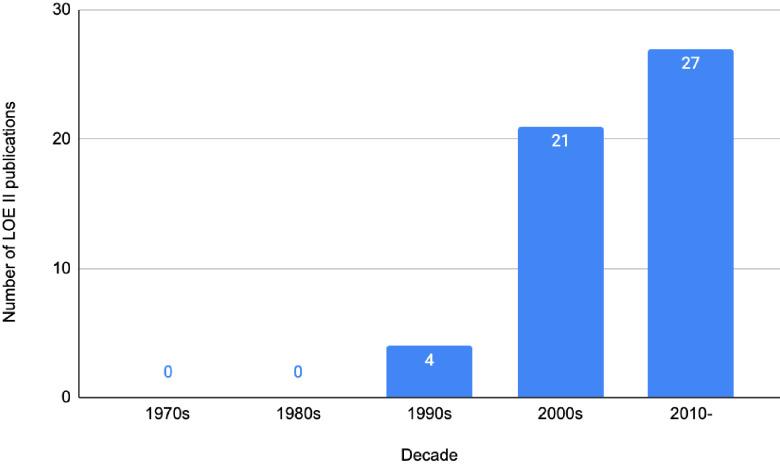
Number of LOE II publications vs. decade within the 100 most cited IBR publications.

Just over half of the publications were assessed to be OCEBM LOE II (*n* = 52), represented by prospective cohort studies (*n* = 45), systematic reviews of non-randomised studies (*n* = 6), and one systematic review and meta-analysis. A total of 41 publications achieved LOE III, whilst 7 achieved LOE IV (**Figure 4**). There were no publications that achieved LOE I or V. The three most utilised research methodologies were prospective cohort studies (*n* = 45), retrospective cohort studies (*n* = 32), and systematic reviews (with no concomitant meta-analysis, *n* = 6). Study designs of the 100 most cited publications are presented in **Figure 5**. Following decade analysis, research productivity, defined as the number of publications, increased over the decades (1970 and 1980s, *n* = 0; 1990s, *n* = 8; 2000s, *n* = 36; 2010–, *n* = 56). Output has increased sevenfold within three decades. Research quality and methodology also improved with each passing decade from the 1990s. Four LOE II publications were published in the 1990s, 21 within the 2000s, and 27 publications from 2010 to the present (**Figures 6**, **7**).

## Discussion

To the best of our knowledge, this is the first bibliometric analysis comprehensively reviewing IBR and QoL measures. Notably, the most cited articles predominantly used the validated BREAST-Q to assess QoL, underscoring its widespread recognition as a psychometrically robust tool, demonstrating accuracy, reliability, and being sensitive to change (7). However, our analysis highlighted several trends and gaps in the literature.

Study designs mainly comprised single-centre prospective cohort studies and case series, thus precluding achievement of the highest LoE. This is not unexpected as patient satisfaction and psychological wellbeing are often assessed on a smaller scale within individual centres, to evaluate the impact of a particular surgical procedure on QoL. Many centres used their own non-validated scales, potentially precluding accurate and reliable data interpretation. Moreover, many retrospective cohort studies did not specify if they used the BREAST-Q pre-operatively in addition to post-operatively, or whether they standardised the timing of questionnaires. Only one study specified they used BREAST-Q post-operatively only. Timing of QoL questionnaires in IBR is important, and a single post-operative BREAST-Q, usually in the early post-operative period, is not sufficient to reflect medium- to long-term complications associated with IBR; serial long-term QoL assessment is required for accurate reflection of the need for future adjustment and revision surgery associated with IBR (14). More recently, the BREAST-Q real-time engagement and communication tool (REACT) has been developed to aid with BREAST-Q score interpretation and guide patient-centred, clinically relevant action recommendations based on longitudinal BREAST-Q scores (15).

In our study, we further assessed the studies utilising the BREAST-Q, which is divided into distinct modules: Augmentation, Reduction/Mastopexy, Mastectomy, Reconstruction, Reconstruction Expectations, and Breast Conserving Therapy. We found that 38 studies employed the BREAST-Q, with many incorporating more than one of these individual modules. Specifically, the “Augmentation” module was used in 1 study, “Breast Conserving Therapy” appeared in 2 studies, “Mastectomy” was utilised in 3 studies, and “Reconstruction” was the most frequently used, appearing in 32 studies. Additionally, “Reconstruction Expectations” was used in one study, and six studies did not specify the module used; however, it is inferred that they most likely utilised the “Reconstruction” module. This distribution indicates a predominant focus on reconstructive outcomes within IBR research.

These findings have significant implications for the interpretation of PROMs. The high prevalence of the “Reconstruction” module highlights the emphasis on reconstructive procedures and patient expectations post-surgery, which can greatly influence the reported satisfaction and QoL. Conversely, the limited use of the “Augmentation” and “Breast Conserving Therapy” modules suggests a potential underrepresentation of these patient groups in the literature at least within the subsection of IBR. Understanding these nuances is crucial for accurately interpreting PROMs and for the development of comprehensive patient care strategies that address the specific needs and expectations of all breast surgery patients.

These findings are consistent with a bibliometric analysis completed by Miller et al., evaluating the 50 most cited breast reconstruction publications between 2000 and 2010; although they focussed on all autologous and implant-based surgical techniques in breast reconstruction primarily assessing surgical outcomes, one single study made it to their list that included PROMs (16). Unlike Miller et al, we did not limit the study time frame for our search to capture all relevant trends from studies published in any year, and focussed purely on PROMs associated with IBR to understand IBR QoL studies in further depth. However, they also noted a lack of high-quality evidence to guide surgical decision-making in the face of increasing surgical options (16). This is even more so for psychological wellbeing assessment, which was usually a secondary outcome in many initial landmark studies on IBR. However, we found many prospective cohort studies, which, as a tool for studying this subject, are valuable and not to be underestimated as suitable evidence for progress in the field.

Whilst the BREAST-Q remains a widely used tool, few studies assessed patients’ psychological wellbeing using validated psychiatric tools like Hospital Anxiety and Depression Scale (HADS) (*n* = 6) or Depression Anxiety Stress Scale (DASS)-21 (*n* = 2); interestingly, the most cited research in our analysis used the HADS tool (13, 17, 18). Moreover, our analysis shows that the presence of a formal existing psychiatric diagnosis was evaluated only in the study by Hopwood and colleagues (19). This represents a gap in the literature, considering that a psychiatric diagnosis can significantly impact QoL and PROs in patients undergoing IBR (20, 21). Validated tools such as the BREAST-Q may assess symptom domains that may be more sensitive to the impact of a psychiatric diagnosis but cannot discriminate the presence of formal psychiatric diagnoses such as major depression, generalised anxiety disorder, or body dysmorphic disorder (21).

Volume replacement and displacement oncoplastic techniques that facilitate breast conservation achieve superior QoL measures compared to simple or flat mastectomy, but significant heterogeneity and the lack of a unified questionnaire preclude meaningful comparison of BCS with mastectomy associated with breast reconstruction, including IBR (22, 23). Although it is recognised that pre–pectoral is better for pain, and aesthetic long–term look with fewer capsular contractures and animation deformities, there is no significant difference in QoL based on current available data, and this did not make it to the 100 most cited articles (24).

Similarly, studies have shown that there is no significant difference in the QoL between patients undergoing DTI reconstruction and those undergoing Two–Stage Reconstruction with Tissue Expanders, notwithstanding the exception of sexual wellbeing, where DTI patients fared better than the tissue expander, implant cohort (25, 26).

The UK–wide Get It Right First Time report recommends routine collection and reporting of PROMs in all breast reconstructive cases pre– and post–operatively (25). The Oncoplastic Breast Consortium also recommends the use of BREAST–Q amongst other PROMs and quality indicators of surgical morbidity (27). Therefore, universal adoption of PROMs is warranted in future IBR studies to enable effective informed consent and decision–making, it is also an important governance tool if standardised as a measure across a country and/or globally. Majority of the studies in our analysis are USA–based. The American breast units have a high–volume practice with a focus on long–term QoL measures supported by financial and healthcare resources, the majority of their studies are led by plastic surgeons who perform IBR, unlike the UK where practice is shared between plastics and breast oncological surgeons. Mastectomy rates are also higher in the USA compared to the UK and western Europe, in turn leading to more patients with IBR, and this may be reflected in the number of studies led by them (28, 29). Nevertheless, future research should encompass global representation, with diverse patient populations and practices. Plastic surgery journals contributed the vast majority of the most cited research, consolidating practice trends, whilst offering insight into how reconstruction improves QoL.

Despite the methodological robustness with which our analysis was conducted, there are few limitations, some inherent to bibliometric analyses. Citation choice can be subject to citation bias, distortion, amplification, and invention, which may result in the unfounded authority of certain publications (30). Importantly, neither LoE nor citation count equates to a study’s overall quality but may serve as a proxy for clinical impact resulting in potential change of practice. Lower LoE studies may add to future systematic reviews’ and meta–analyses’ databases and thereby contribute to the evidence base in the field. The impact of IBR on QoL would be subject to response and selection bias, especially if retrospective. Breast implant–associated lymphoma, although rare, has an impact on wellbeing as does the challenging, unclear diagnosis of breast implant illness (BII), these studies are not highly cited but crucial (31–33).

Despite these limitations, an extensive search of the literature was conducted and the articles presented here may be considered seminal in IBR and QoL. For future studies in this area, we recommend better–designed prospective studies that assess serial long–term QoL measures with validated tools. In the current economic climate, we must value the financial burden of IBR, and therefore, QoL is an important measure to sustain practice in terms of resource allocation prioritisation for otherwise seemingly high–cost elective surgery.

## Conclusion

This comprehensive bibliometric analysis comprehensively examines the top 100 most highly cited publications regarding QoL in IBR and shows the evolution of trends in the field over the past five decades. Research areas that are materialising within this field entail assessing and optimising patient satisfaction and psychological wellbeing via validated patient questionnaires. Despite the quality of literature being reasonably high, more emphasis can be placed on undertaking the publication of methodologically robust studies with higher OCEBM LOE, such as well–designed RCTs or multicentre prospective studies with serial long–term QoL measures with validated tools. Additionally, the development and adoption of more validated PROMs designed for IBR is centrally important for calibrating patient satisfaction with clinical outcomes and providing a noticeable greater QoL following IBR.

## Data availability statement

The original contributions presented in the study are included in the article/**Supplementary Material**. Further inquiries can be directed to the corresponding author.

## Author contributions

KD: Writing – review & editing, Writing – original draft, Visualization, Validation, Software, Methodology, Investigation, Formal analysis, Data curation. FR: Writing – review & editing, Writing – original draft, Validation, Supervision, Software, Project administration, Methodology, Investigation. RM: Writing – review & editing, Writing – original draft, Validation, Supervision, Project administration, Methodology. TB: Writing – review & editing, Writing – original draft, Visualization, Project administration, Methodology. RA: Writing – review & editing, Writing – original draft, Validation, Data curation. MC: Writing – original draft, Writing – review & editing, Supervision, Conceptualization. AK: Validation, Writing – original draft, Writing – review & editing, Supervision, Resources, Project administration, Conceptualization.

## Supplementary material

Supplementary Table 1 Search strategy.

Supplementary Table 2 The 100 most cited publications regarding IBR.

Supplementary Table 3 Journal frequency in the 100 most cited IBR publications.

Supplementary Table 4 Frequency of quality-of-life measures/PROs.
