# Supplementary material for: Bibliometric analysis of quality of life in implant-based breast reconstruction
Source: Front Oncol. 2024 Aug 8;14:1429885. doi: 10.3389/fonc.2024.1429885 (PMC11339687; doi:10.3389/fonc.2024.1429885)
Supplement: Supplementary Table 1 — Search strategy. [file Table_1.docx]

| Stem 1 | "PRO?*" OR "patient reported outcome*" OR "patient reported outcome measure" OR "personal satisfaction" OR "outcome assessment" OR "QoL" OR "quality-of-life" OR "mental wellbeing" OR "psychological wellbeing" OR "mental health survey" OR "breast q" OR "breast-q" OR "PROM" OR "PROMs" or "mental health outcomes" or "core 30" or "breast 23" and "implant-based" or "implant reconstruction" or "breast reconstruction" or "implant-based reconstruction" or "PMRT" or "post implant mastectomy" or "post implant mastectomy radiotherapy" or "implant radiotherapy" or "radiotherapy with implant" or "implant with radiotherapy" or "implant without radiotherapy" or "irradiation with implant" or "irradiation with breast implant" or "pre-pectoral implant" or "sub-pectoral implant" or "with ADM" or "without ADM" |
| --- | --- |
