# Supplementary material for: Bibliometric analysis of quality of life in implant-based breast reconstruction
Source: Front Oncol. 2024 Aug 8;14:1429885. doi: 10.3389/fonc.2024.1429885 (PMC11339687; doi:10.3389/fonc.2024.1429885)
Supplement: Supplementary Table 2 — The 100 most cited publications regarding IBR. [file Table_2.docx]

| Rank | Author | Corresponding Author | Citations | Mean citations per year |
| --- | --- | --- | --- | --- |
| 1 | Al-Ghazal, SK et al | Al-Ghazal, SK | 457 | 18.28 |
| 2 | Wilkins, EG et al | Wilkins, EG | 372 | 14.88 |
| 3 | Alderman, AK et al | Alderman, AK | 304 | 12.16 |
| 4 | Krueger, EA et al | Pierce, LJ | 300 | 12.5 |
| 5 | Yueh, JH et al | Lee, BT | 293 | 19.53 |
| 6 | Elder, EE et al | Elder, EE | 286 | 14.3 |
| 7 | Cordeiro, PG et al | Cordeiro, PG | 278 | 13.24 |
| 8 | Atisha, D et al | Wilkins, EG | 261 | 15.35 |
| 9 | Santosa KB et al | Pusic AL | 244 | 34.86 |
| 10 | Hu, ES et al | Alderman, AK | 241 | 15.06 |
| 11 | Eltahir, Y et al | Eltahir, Y | 226 | 18.83 |
| 12 | Pusic, AL et al | Pusic, AL | 209 | 26.13 |
| 13 | Harcourt, DM et al | Harcourt, DM | 209 | 9.5 |
| 14 | Schover, LR et al | Schover, LR | 199 | 6.63 |
| 15 | Cordeiro, PG and McCarthy, CM | Cordeiro, PG | 195 | 10.26 |
| 16 | Ascherman, JA et al | Ascherman, JA | 191 | 10.05 |
| 17 | Jagsi, R et al | Jagsi, R | 177 | 17.7 |
| 18 | Sheehan, J et al | Sherman, KA | 172 | 9.56 |
| 19 | Jagsi, R et al | Jagsi, R | 156 | 22.29 |
| 20 | Bengtson, BP et al | Bengtson, BP | 147 | 8.17 |
| 21 | Nano, MT et al | Nano, MT | 146 | 7.3 |
| 22 | Atisha, DM et al | Atisha, DM | 145 | 14.5 |
| 23 | Morrow, M et al | Morrow, M | 144 | 13.09 |
| 24 | Haloua, MH et al | van den Tol, MP | 143 | 11.92 |
| 25 | Arora, NK et al | Arora, NK | 143 | 5.96 |
| 26 | Wei, CH et al | Pusic, AL | 142 | 15.78 |
| 27 | Cordeiro, PG et al | Cordeiro, PG | 142 | 14.2 |
| 28 | Chen, CM et al | Pusic, AL | 140 | 9.33 |
| 29 | Lodder, LN et al | Lodder, LN | 137 | 5.96 |
| 30 | Yurek, D et al | Andersen, BL | 136 | 5.44 |
| 31 | Alderman, AK et al | Alderman, AK | 133 | 7.39 |
| 32 | Nissen, MJ et al | Nissen, MJ | 133 | 5.54 |
| 33 | Clough, KB et al | Clough, KB | 132 | 5.5 |
| 34 | Caffo, O et al | Caffo, O | 130 | 5.91 |
| 35 | Andrade, WN et al | Semple, JL | 128 | 5.33 |
| 36 | Stefanek, ME et al | Stefanek, ME | 128 | 4.27 |
| 37 | Howes, BHL et al | Dean, NR | 127 | 14.11 |
| 38 | Cordeiro, PG et al | Cordeiro, PG | 127 | 11.55 |
| 39 | Serra-Renom, JM et al | Serra-Renom, JM | 126 | 8.4 |
| 40 | Nelson, JA et al | Nelson, JA | 116 | 19.33 |
| 41 | Frasier, LL et al | Neuman, HB | 116 | 12.89 |
| 42 | Rowland, JH et al | Rowland, JH | 114 | 3.56 |
| 43 | Colakoglu, S et al | Lee, BT | 111 | 7.93 |
| 44 | Lee, C et al | Lee, C | 110 | 6.88 |
| 45 | [Bailey, CR](https://www-webofscience-com.sheffield.idm.oclc.org/wos/author/record/4639060) et al | Rosson, GD | 108 | 13.5 |
| 46 | Yoon, AP et al | Wilkins, EG | 107 | 15.29 |
| 47 | Toyserkani, NM et al | Toyserkani, NM | 106 | 21.2 |
| 48 | Kagawa-Singer, M et al | Kagawa-Singer, M | 106 | 3.79 |
| 49 | McCarthy, CM et al | McCarthy, CM | 102 | 6.8 |
| 50 | Veiga, DF et al | Veiga, DF | 102 | 6.8 |
| 51 | Hopwood, P et al | Hopwood, P | 102 | 4.08 |
| 52 | Salgarello, M et al | Salgarello, M | 101 | 7.77 |
| 53 | Girotto, JA et al | Nahabedian, MY | 97 | 4.41 |
| 54 | Walia, GS et al | Sacks, JM | 92 | 13.14 |
| 55 | Buchanan, PJ | Momoh, AO | 92 | 4.38 |
| 56 | Baker, BG et al | Baker, BG | 91 | 13 |
| 57 | Guyomard, V et al | Leinster, S | 91 | 5.06 |
| 58 | Cocquyt, VF et al | Cocquyt, VF | 90 | 4.09 |
| 59 | Winters, ZE et al | Winters, ZE | 89 | 5.93 |
| 60 | Liu, CJ et al | Lee, GK | 88 | 8 |
| 61 | [Dauplat, J](https://www-webofscience-com.sheffield.idm.oclc.org/wos/author/record/20857491) et al | Pomel, C | 87 | 10.88 |
| 62 | Kronowitz, SJ et al | Kronowitz, SJ | 87 | 6.69 |
| 63 | Spear, SL et al | Spear, SL | 87 | 5.12 |
| 64 | Bresser, PJC et al | Bresser, PJC | 86 | 4.53 |
| 65 | Cederna, PS et al | Cederna, PS | 85 | 2.83 |
| 66 | Fanakidou, I et al | Alikari, V | 84 | 12 |
| 67 | lbornoz, CR et al | Pusic, AL | 84 | 7.64 |
| 68 | Metcalfe, KA et al | Metcalfe, KA | 84 | 6.46 |
| 69 | Craft, RO et al | Lee, BT | 84 | 5.13 |
| 70 | Ramon, Y et al | Ramon, Y | 84 | 3 |
| 71 | Fang, SY et al | Chang, YJ | 83 | 6.92 |
| 72 | Pusic, AL et al | Pusic, AL | 82 | 6.31 |
| 73 | Saulis, AS et al | Fine, NA | 82 | 4.56 |
| 74 | Nicholson, RM et al | Nicholson, RM | 81 | 4.5 |
| 75 | Voineskos, SH et al | Voineskos, SH | 80 | 16 |
| 76 | Ashraf, AA et al | Lee, BT | 80 | 6.67 |
| 77 | McCarthy, CM et al (pusic 2nd author) | Cordeiro PG | 80 | 4 |
| 78 | Fernandez-Delgado, J et al | Reza, MM | 79 | 4.65 |
| 79 | Rosenqvist, S et al | Sandelin, K | 78 | 2.69 |
| 80 | Eltahir, Y et al | Eltahir, Y | 77 | 7.7 |
| 81 | Heneghan, HM et al | Heneghan, HM | 77 | 5.5 |
| 82 | Pusic, AL et al | Kerrigan, CL | 77 | 2.96 |
| 83 | Eriksson, M et al | de Boniface, J | 76 | 6.33 |
| 84 | Snell, L et al (pusic 2nd author) | Pusic, AL | 75 | 5 |
| 85 | Matros, E et al (pusic 2nd author) | Matros, E | 74 | 7.4 |
| 86 | Zhong, T et al | Zhong, T | 73 | 6.08 |
| 87 | Macadam, SA et al (pusic 2nd author) | Macadam, SA | 73 | 4.87 |
| 88 | Isern, AE et al | Isern, AE | 73 | 4.29 |
| 89 | Roth, RS et al | Roth, RS | 73 | 3.65 |
| 90 | Koslow, S et al | Koslow, S | 72 | 6 |
| 91 | Pirro, O et al | Mestak, O | 70 | 9.25 |
| 92 | D'Souza, N et al | D'Souza, N | 70 | 5 |
| 93 | Tønseth, KA | Tønseth, KA | 70 | 4.18 |
| 94 | Kelsall, JE et al | Kelsall, JE | 69 | 8.63 |
| 95 | Macadam, SA et al | Macadam, SA | 68 | 5.67 |
| 96 | Romanoff, A et al | Morrow, M | 67 | 9.57 |
| 97 | Zehra, S et al | Zehra, S | 64 | 12.8 |
| 98 | Susarla, SM et al | Chun, YS | 64 | 6.9 |
| 99 | Gui, GPH et al | Gui, GPH | 64 | 2.95 |
| 100 | Lagendijk, M et al | Lagendijk, M | 63 | 9.14 |

Table 1 – 100 most cited publications on patient satisfaction and psychological well-being in implant-based reconstruction.
