# Supplementary material for: Bibliometric analysis of quality of life in implant-based breast reconstruction
Source: Front Oncol. 2024 Aug 8;14:1429885. doi: 10.3389/fonc.2024.1429885 (PMC11339687; doi:10.3389/fonc.2024.1429885)
Supplement: Supplementary Table 3 — Journal frequency in the 100 most cited IBR publications. [file Table_3.docx]

| Rank | Source Journal | Number of Publications | Impact Factor |
| --- | --- | --- | --- |
| 1 | Plastic and Reconstructive Surgery | 39 | 3.6 |
| 2 | Journal of Plastic Reconstructive and Aesthetic Surgery | 6 | 2.7 |
| 3 | Breast Cancer Research and Treatment | 5 | 3.8 |
| 4 | Annals of Surgery | 5 | 10.1 |
| 5 | Annals of Surgical Oncology | 4 | 3.7 |
| 6 | Cancer | 4 | 6.2 |
| 7 | The Breast | 4 | 3.9 |
| 8 | European Journal of Surgical Oncology | 3 | 3.8 |
| 9 | Journal of the American College of Surgeons | 3 | 5.2 |
| 10 | Psycho-Oncology | 3 | 3.6 |
| 11 | Annals of Plastic Surgery | 2 | 1.5 |
| 12 | JAMA Surgery | 2 | 16.9 |
| 13 | Plastic and Reconstructive Surgery Global Open | 2 | 1.5 |
| 14 | The Breast Journal | 2 | 3.9 |
| 15 | ANZ Journal of Surgery | 1 | 1.7 |
| 16 | Annals of Oncology | 1 | 50.5 |
| 17 | Breast Cancer | 1 | 4 |
| 18 | British Journal of Surgery | 1 | 9.6 |
| 19 | Cochrane Database of Systematic Reviews | 1 | 8.4 |
| 20 | Culture Medicine and Psychiatry | 1 | 1.7 |
| 21 | European Journal of Cancer | 1 | 8.4 |
| 22 | Expert Review of Pharmacoeconomics & Outcomes Research | 1 | 2.3 |
| 23 | International Journal of Radiation Oncology - Biology - Physics | 1 | 7 |
| 24 | Journal of Clinical Oncology | 1 | 45.3 |
| 25 | Journal of Consulting and Clinical Psychology | 1 | 5.9 |
| 26 | Journal of Surgical Research | 1 | 2.2 |
| 27 | Journal of the National Cancer Institute | 1 | 10.3 |
| 28 | Preventive Medicine | 1 | 5.1 |
| 29 | Psychosomatics | 1 | 3.4 |
| 30 | Quality of Life Research | 1 | 3.5 |
