# Supplementary material for: Bibliometric analysis of quality of life in implant-based breast reconstruction
Source: Front Oncol. 2024 Aug 8;14:1429885. doi: 10.3389/fonc.2024.1429885 (PMC11339687; doi:10.3389/fonc.2024.1429885)
Supplement: Supplementary Table 4 — Frequency of quality-of-life measures/PROs. [file Table_4.docx]

| Rank | Quality of life measures/questionnaires | Frequency |
| --- | --- | --- |
| 1 | Self-assessment/customised questionnaire | 69 |
| 2 | BREAST-Q | 38 |
| 3 | Body Image after Breast Cancer Questionnaire (BIBCQ) | 12 |
| 4 | Functional Assessment of Cancer Therapy-Breast (FACT-B) | 10 |
| 5 | EORTC QLQ-BR23 | 9 |
| 6 | Short-Form 36 (SF-36) | 7 |
| 7 | Hospital Anxiety Depression Scale (HADS) | 7 |
| 8 | Body Image Scale 10 (BIS-10) | 6 |
| 9 | Michigan Breast Reconstruction Outcomes Study (MBROS) Self-assessment questionnaire | 6 |
| 10 | Rosenberg Self Esteem Scale (RSE) | 4 |
| 11 | EORTC QLQ-C30 | 4 |
| 12 | BIS-10 | 4 |
| 13 | Depression Anxiety and Stress Scale 21 (DASS-21) | 3 |
| 14 | DAS-59 | 3 |
| 15 | Hopwood BIS | 3 |
| 16 | HDA | 2 |
| 17 | Likert scale | 2 |
| 18 | SF-12v2 | 2 |
| 19 | RAND-36 | 2 |
| 20 | PROMIS-29 | 3 |
| 21 | Decision Regret Scale | 2 |
| 22 | Mastectomy Attitude Scale | 2 |
| 23 | Sexual Activity Questionnaire | 2 |
| 24 | Medical Outcomes Study Short Form | 2 |
| 25 | Impact of Event Scale | 2 |
| 26 | Concerns About Recurrence Scale (CARS) | 1 |
| 27 | Psychosocial Adjustment to Illness Scale Self-Report Form (PAIS-SR) | 1 |
| 28 | Body Esteem Scale (BES) | 1 |
| 29 | Satisfation with Reconstructive Outcomes | 1 |
| 30 | Satisfaction w Breast Cosmetic Outcomes | 1 |
| 31 | Positive and Negative Affect Schedule | 1 |
| 32 | Miller Behavioural Style Scale | 1 |
| 33 | Functional Assessment of Cancer Therapy-General (FACT-G) | 1 |
| 34 | General Breast Cancer Quality of Life | 1 |
| 35 | Breast Cancer Body Image | 1 |
| 36 | Polivy BIS | 1 |
| 37 | Breast Cancer Treatment Outcome Scale (BCTOS) | 1 |
| 38 | Sexual Self Schema Scale for Women | 1 |
| 39 | Breast Impact of Treatment Scale (BITS) | 1 |
| 40 | Body Satisfaction Scale (BSS) | 1 |
| 41 | Mischel Uncertainty in Illness Scale | 1 |
| 42 | Profile of Mood States | 1 |
| 43 | Short Form-McGill Pain questionnaire | 1 |
| 44 | McCormick Sensitive Questionnaire | 1 |
| 45 | Visual Analogue Scale | 1 |
| 46 | Body Cathexis Scale | 1 |
| 47 | HAM-A | 1 |
| 48 | HAM-D | 1 |
| 49 | CARES | 1 |
| 50 | ASSIS | 1 |
| 51 | ISSB, | 1 |
| 52 | Suinn-Lew Self-Identity Acculturation Scale | 1 |
| 53 | Weiner-Adler Health Symptoms | 1 |
| 54 | CES-D | 1 |
| 55 | AABCSQ | 1 |
| 56 | Qualitative Questionnaire of Breast Cancer Experience: Kleinman’s Explanatory Model | 1 |
| 57 | GHQ-28 | 1 |
| 58 | MOS-36 | 1 |
| 59 | UCLA | 1 |
| 60 | MVQOLI-15 | 1 |
| 61 | Quality of Life Index | 1 |
| 62 | Brief Symptom Inventory | 1 |
| 63 | HR-QoL | 1 |
| 64 | Modified Stanford Self-Efficacy Scale | 1 |
| 65 | HBIS | 1 |
| 66 | ROMIS-29 | 1 |
